# Vaccine Virus

**Published:** 1883-02

**Authors:** 


### EDITORIAL, ETC.

*Vaccine Virus.*—How it is obtained by the Innoculation of heifers, and the preparation of the lymph for use :

In view of the prevalence of small pox throughout the country and the grossly exaggerated reports concerning the extent of the diseases in Baltimore, where it is now confined to limited areas in the Southern and Eastern portions of the city, and even there rapidly decreasing, the following description will prove instructive :

Taking the Washington City national vaccine establishment for an example : At present the stock on hand numbers sixty heifers, ranging from six to twelve months of age. These heifers are mostly thoroughbred, with fine skins and soft hair. Dr. Walsh never vaccinates a scrub calf if he can help it. After being brought to the farm they are carefully stalled and fed for a week or ten days before vaccination. From the farm, which is some distance from Washington, they are brought to the establishment in lots ranging in number according to the demand for virus, and kept under the personal supervision of Dr. Walsh until the process of vaccination and collection of the virus is complete.

The process is as follows : The heifer is carefully strapped on her back on a padded trough, under and adjacent parts of the abdomen are smoothly shaved. The scarifications for putting in the virus vary in number according to the size of the heifer, generally about a dozen points of vaccination, each being three-quarters of an in inch in diameter. This vaccination is done with seed virus propagated from heifer to heifer, and is of the Beaugency stock. The virus originally comes from a disease peculiar to cows known as cow-pox. After the vaccinaiion an apron is placed over the vaccinated surface as a protection against dirt. The effect on the calf is hardly noticeable. Food is taken as usual, and up to the sixth day there is no striking change in the vaccinated places. By that time the vesicles are filled, and the virus is taken from the sixth to the seventh day. Pure vaccine lymph is colorless; a red tint indicates the presence of blood. When a quill or point presents a red or brown tint it is due to the presence of blood; a yellow tint indicates the presence of pus, each of which is a foreign and possibly dangerous material. Nothing but quill slips are used by this establishment.

The quills are boiled to rid them of any grease, then filed, split and pointed. They are charged with the virus by dipping the convex surface of the pointed end in the lymph as it exudes from the vesicle. In order to do this the calf is strapped on its back on the trough on which it was vaccinated. Here is where honesty and dishonesty may be shown in this business. A vesicle yields but a certain amount of pure lymph. After it has been removed on the surface of these quills, or points, serum will commence to well up from the bottom of the vesicle. This serum is feebly charged with virus and should not be furnished as a genuine article. The careful collector of this virus stands bent over the heifer for about four hours, dipping the quills in the exuding virus. The heifers are held in a comfortable position by straps, loose enough to allow them the privilege of kicking a little. After four hours the animals begin te experience some discomfort from their unnatural position, and are put on their feet again for a time at least.

After being charged the quills are carefully dried in a cool place before being packed. They are wrapped in packages of ten, first with tissue paper, then rubber tissue, inclosed in a tightly-corked vial, which is placed in a metal mailing package for transportation. The number of quills taken from a heifer is a lottery, sometimes not as much virus being produced as was put on in vaccinating. In exceptional cases 2,500 points have been taken from a heifer. A heifer can never be used more than once for the purpose, her constitution being changed by the process. The vaccination not only does not hurt the heifer, but is a positive benefit. The animal, after the process, improves more rapidly, fills out and develops. It has been clearly shown in northern herds that it acts as a preventive of contagious diseases. In herds attacked with pleuro-pneumonia and other contagious diseases, those animals that had been vaccinated escaped. Dr. Walsh believes that any disrepute into which bovine virus may have fallen is the result of dishonesty practiced in its preparation. The pure lymph in his opinion is as active to-day as when the Beaugency stock was first procured. Dr. Walsh frequently receives orders for the vaccine crusts off the heifers, but he discourages such use of them, as he does not think them reliable.

Dr. F. M. Welles, one of the proprietors of the New England vaccine establishment, who is in the city says that their business at Chelsea, Mass., has been in existence 12 years. Last year 1,976,000 points charged with virus were sent to various parts of this country, South America, West Indies, China, &c. The doctor said that none but the best stock is used, animals being selected which have thin skin and short hair. They are placed in stables and fed only on hay, grain being considered too heating. Well-grown cattle, fat enough for beef, are considered the best. The bone or ivory points are first dipped in the lymph from one animal, allowed to dry, and then dipped in that coming from another animal. Dr. Welles says it requires double the amount of lymph to charge an ivory point that will charge a quill. The virus, before being used is put to the highest test as to purity, and care is taken that no blood gets upon the points, for fear that blood-poisoning might be produced.

